# Recent findings on the health effects of omega-3 fatty acids and statins, and their interactions: do statins inhibit omega-3?

**DOI:** 10.1186/1741-7015-11-5

**Published:** 2013-01-04

**Authors:** Michel de Lorgeril, Patricia Salen, Pascal Defaye, Mikael Rabaeus

**Affiliations:** 1 Laboratoire Cœur et Nutrition, TIMC-IMAG CNRS 5525, Université Joseph Fourier, Faculté de Médecine de Grenoble, 38054 La Tronche, France; 2 Service de Cardiologie, Hôpital Universitaire Nord, 38054 La Tronche, France; 3 Clinique La Prairie, Chemin de la Prairie 2-10, 1815 Clarens, Montreux, Switzerland

**Keywords:** omega-3 fatty acids, statins, ischemic heart disease, myocardial infarction, randomized clinical trials, epidemiology, mitochondria, insulin resistance, diabetes, n-6 fatty acids

## Abstract

Early randomized controlled trials (RCTs) demonstrated the health benefits of omega-3 fatty acids (n-3), whereas recent RCTs were negative. We now address the issue, focusing on the temporal changes having occurred: most patients in recent RCTs are no longer n-3 deficient and the vast majority are now treated with statins. Recent RCTs testing n-3 against arrhythmias suggest that n-3 reduce the risk only in patients not taking a statin. Other recent RCTs in secondary prevention were negative although, in a *post-hoc *analysis separating statin users and non-users, non-significant protection of n-3 was observed among statin non-users whereas statin users had no effect. Recent RCTs testing statins - after the implementation of the New Clinical Trial Regulation in 2007 - are negative (or flawed) suggesting that the lack of effect of n-3 cannot be attributed to a parallel protection by statins. Finally, statins favor the metabolism of omega-6 fatty acids (n-6), which in turn inhibits n-3 and, contrary to n-3, they increase insulin resistance and the risk of diabetes. Thus, n-3 and statins are counteractive at several levels and statins appear to inhibit n-3.

## Introduction

Until 2005, studies consistently provided clear evidence that omega-3 fatty acids (n-3) protect against cardiovascular diseases (CVD) complications [1,2]. They were thought to reduce the risk of arterial atherosclerotic and thrombotic obstruction [3,4]; to increase the myocardial resistance to ischemia-reperfusion injury [5,6]; and to prevent malignant ventricular arrhythmias [7-9]. Animal [5-7] and epidemiological studies [8,9] as well as randomized controlled trials (RCTs) [10-13] all supported that n-3 are protective. This was confirmed in meta-analyses of both prospective cohort studies and RCTs leading to the conclusion that an intake of 250 mg/day of marine n-3 (EPA+DHA (eicosapentanoic acid + docosahexanoic acid)) reduced fatal CVD by 36% when compared to no EPA+DHA [14].

Consequently, it was proposed to use blood measurements of n-3 as a predictor of CVD complications. The omega-3 index - defined as the percentage of EPA+DHA in blood red cells [15] - reflects the average dietary intake and the tissue levels of EPA+DHA, including those of the heart [16,17]. A high omega-3 index (> 8%) is thought to be associated with a low risk of CVD complications [15] whereas a low omega-3 index (< 4%) is associated with increased risk susceptible to be decreased by a preventive treatment with n-3 (fish oil) supplements. An omega-3 index between 4 and 8% indicates an intermediate risk. The effects of n-3 supplements are, therefore, expected to be different in patients with either high or low omega-3 index with large benefits for those with a low index (that is, high risk) and small or no benefits for those with a high index (that is, low risk). This concept is critical because it suggests that n-3 supplements might be potentially protective against CVD complications only in patients who are n-3 deficient and not in patients who are at high risk for reasons other than an n-3 deficiency. This underlines the fact that n-3 are nutrients and not a drug. So far this concept applies only for CVD, not for other nonvascular and non-cardiac clinical conditions.

Contrary to the expectations, the most recent RCTs - that is, those published after 2005 - did not confirm the protective action of n-3 [18-23]. In a recent meta-analysis examining the efficacy of n-3 supplements (EPA+DHA) in the secondary prevention of coronary heart disease (CHD), authors analyzed 13 RCTs involving 20,485 patients with a history of CHD and concluded that n-3 supplements did not consistently reduce CHD mortality, all-cause mortality and the risk of overall CVD complications [24]. An explanation could be that the populations enrolled in the most recent RCTs were different from those tested in the past RCTs. Indeed, the authors did not separate early (positive) and recent (negative) RCTs in their meta-analyses and thereby ignored any temporal changes in the dietary and blood n-3 status of the patients enrolled in the early or the recent RCTs. Also, in subgroup analyses by concomitant medication use, the authors report a non-significant preventive effect against the risk of CVD events (relative risk 0.74, 95% confidence intervals 0.54 to 1.03) among patients not receiving statins whereas those receiving statins had no protection at all: relative risk 1.02, 95% confidence intervals 0.92 to 1.12 [24]. These data suggest strong interactions between n-3 and statins and may at least partly explain the discrepancy between recent and early RCTs because the use of statins has become almost systematic among patients in recent RCTs, whereas it was rare or even absent in early RCTs [10-13].

Another puzzling observation is that, contrary to the results of recent RCTs, recent epidemiological studies examining associations between n-3 (or fish intake) and CVD in various populations still demonstrate significant inverse correlation [1,2,25-29]. This further suggests that in populations with low use of statins, n-3 remain apparently protective.

How could these findings be explained?

### Recent RCTs testing the effects of n-3 fatty acids

Among the most recent n-3 RCTs, there are two main categories: those testing whether n-3 reduce the risk of malignant ventricular arrhythmias in patients with an implantable cardiac defibrillator [18-20], and those testing the effects of n-3 in secondary prevention of CHD [21-23]. One trial published in 2003 in secondary prevention (DART-2) was not incorporated in the present analysis because of major design issues [30]. For instance, the trial was interrupted after one year and then re-started with a nonconventional re-randomization (which resulted in different numbers of patients in each of the four groups), there was no true control group, no placebo to compare with the fish oil capsules, and finally a total lack of "blinding" [30]. Another study [31], often cited because the authors reported an increased risk for ventricular arrhythmias among heart failure patients with the highest n-3 concentrations in their red blood cells, was also not incorporated in the present analysis because it is a very short (one year) and very small observational study (n = 102) [31].

Thus, in the first category, we have retained three RCTs.

The first one was published in June 2005 and reported no significant effect of 1.3 g/day of EPA+DHA [18]. The omega-3 index of patients receiving EPA+DHA increased from 4.7 to 8.3% indicating that patients were not n-3 deficient at baseline and were compliant during the follow-up [18]. About half of the patients in both groups were taking a statin. Limitations of the trial were the small sample size (n = 100 per group) and clinical heterogeneity - ischemic vs. non ischemic heart disease - of the underlying cardiac diseases. The second trial was published in November 2005 and reported a non-significant effect (28% risk reduction, *P *= 0.057 in the intention-to-treat analysis) of 2.6 g/day of EPA+DHA [19]. Interestingly, the effect was significant among patients with ischemic heart disease (hazard ratio 0.70, 95% CI 0.45 to 1.00) and also, no patient was taking a statin in either group. The omega-3 index increased from 3.4 to 7.6% in the EPA+DHA group indicating that these patients were slightly n-3 deficient at baseline and compliant. A major limitation was the short follow-up (12 months). The third trial was published in June 2006 and reported no significant effect of 0.96 g/day of EPA+DHA (20). Only 45% of the patients were taking a statin. The short follow-up (12 months) was the main limitation of the study. Measurements of plasma EPA indicated that patients were not n-3 deficient at baseline and were then compliant. There was no protective effect of EPA+DHA in the primary analysis, but a non-significant trend toward protection in the EPA+DHA group among patients with ischemic heart disease: hazard ratio 0.76, 95% CI 0.52 to 1.11 [20].

In summary, these three RCTs do not support a strong protective effect of EPA+DHA against malignant arrhythmias in patients with an implantable cardiac defibrillator (ICD). There are, however, several limitations in each trial: short follow-up, small sample size and medical heterogeneity - ischemic vs. non-ischemic heart disease - of the enrolled patients. Thus, they should be considered individually with precaution. In a subsequent meta-analysis combining the three trials, it was again concluded that EPA+DHA are not protective [32]. However, the effect among patients with ischemic heart disease (hazard ratio 0.79, 95% CI 0.60 to 1.06) tended toward protection. It has to be noted that the pooled sample size remained small suggesting that the meta-analysis itself was underpowered to detect protection in patients with ischemic heart disease. Finally, in a *post-hoc *analysis combining the two trials in which statins were prescribed to some patients (n = 333), a significant interaction (*P *< 0.05) was noted between statins and n-3 suggesting that the association of statins with n-3 may reduce the benefits of each treatment [32]. Indeed, in the trial with the most favorable effect of n-3, no patient was taking a statin [19]. While this interaction, also reported in the meta-analysis discussed above [24], might be a chance finding, it becomes critical to address the issue in future studies. Overall then, these recent RCTs [18-20] suggest that among patients not severely n-3 deficient at baseline, n-3 supplements may reduce the risk of malignant arrhythmias, but only among patients with ischemic heart disease and not taking statins. This would reach agreement with early RCTs and current knowledge on n-3 and CVD complications [1-14].

Finally, we note that some recent experimental studies also did not confirm the anti-arrhythmic effects of n-3 previously reported [7-10] and may even have detected an arrhythmogenic effect [33,34]. In one study, the main difference from previous studies using a similar model of ischemia-induced arrhythmias [7] was that n-3 were given orally in the form of ethyl esters in the recent studies [33], instead of through intravenous infusion of purified n-3 [7]. Whether the dietary administration itself (vs. infusion) or the use of quite large doses of ethyl esters (rather than purified n-3) explains these recent data deserves further investigation. Some other experimental studies were also confusing and difficult to interpret. For instance, the same group of investigators published, the same year 2007, data showing either promotion of arrhythmias by n-3 in isolated pig hearts [34] or reduction of the incidence of arrhythmias by n-3 in pig ventricular myocytes [35].

In any case, compared with previous human studies, a new RCT testing n-3 supplements would require a larger sample size, longer follow-up and selection of patients with ischemic heart disease - with and without statins - and a low omega-3 index at baseline. As a lab to lab variability in n-3 measurements may confuse the data, these measurements should be done in a unique central lab. As the probability of such a RCT is very low, a careful analysis of the recent RCTs in secondary prevention of CHD is mandatory.

Regarding this second category of trials - RCTs testing n-3 in secondary prevention of CHD - one should consider two periods: before and after the systematic use of statins in secondary prevention of CHD. During the pre-statin period, the mid-1980s to the end of 1990s, two large trials (DART and GISSI), reported significant benefits of marine n-3 in survivors of a recent myocardial infarction [10,11]. On the basis of these two trials, and also of the results of the Lyon Diet Heart Study (in which patients were supplemented with the plant n-3 alpha-linolenic acid, the precursor of EPA and DHA in the endogenous pathway [36,37], leading to a significant increase in EPA), n-3 were claimed to reduce the risk of CHD complications and mortality in secondary prevention [14]. Importantly, few patients were taking statins and their average intake of n-3 was low. These three trials had a large media coverage and the idea that plant and marine n-3 are critical for the prevention of CHD spread rapidly to the medical community and the public. In 2002, the American Heart Association recommended two fatty fish meals per week for the general population and 1 g EPA+DHA per day for patients with CHD [38]. As a consequence, during the next period, after the year 2000 approximately, the vast majority of CHD patients, in addition to taking statins, were aware of the importance of having more n-3 in their diet leading many survivors of a heart attack to take an n-3 supplement. Thus, the conditions in which n-3 supplements were tested in secondary prevention in this second period [21-23] were very different from those of the first one [10-13].

If we only consider RCTs reporting 'hard' (myocardial infarction and cardiac death) endpoints, with sample size and follow-up large enough to analyze mortality, three RCTs should be examined.

In a first RCT (Sufolom3), 2,501 CHD patients were randomized to receive either 0.6 g/day of EPA+DHA or placebo and were followed up for 4.7 years [21]. About 85% of patients were taking a statin. Baseline plasma n-3 levels were high, indicating that most patients were not n-3 deficient. For instance, plasma EPA at baseline (1.20% of total fatty acids) was higher than the level measured in the experimental group of the Lyon Trial (1.03 ± 0.06 vs. 0.76 ± 0.05% in the control group) receiving n-3 supplementation [12,13]. In Sufolom3, allocation to n-3 was not associated with any significant benefit. A limitation of the trial was the low complication rate (1.3% per year) compared, for instance, with 4.9% in the Lyon trial.

In a second RCT (Omega), 3,851 survivors of a recent myocardial infarction were given 0.84 g/day of EPA+DHA (compared with a placebo) [22]. About 95% of the patients were taking a statin and the consumption of n-3 was rather high as only 3% of patients were not eating fish and about half of the patients were eating fish several times a week. There was no significant difference between the patients receiving n-3 and those taking the placebo [22]. A major limitation of the trial was the short follow-up (one year).

In a third trial (Alpha Omega), 4,837 patients who had a myocardial infarction were randomized to receive for 40 months one of four margarines: a margarine supplemented with EPA+DHA, a margarine supplemented with ALA, a margarine supplemented with EPA+DHA+ALA and a placebo margarine [23]. On average, patients consumed 19 g of margarine per day, which resulted in additional daily intakes of 380 mg EPA+DHA, 1.9 g ALA or both in the active-treatment groups. About 86% of patients were taking a statin and average consumption of fish at baseline was 15 g/day in each subgroup corresponding to an average intake of EPA+DHA of about 125 mg/day, which was confirmed by measuring blood n-3. Treatment with n-3 did not reduce the risk in the primary analysis whereas some benefits, in subgroup analyses, could be seen in women and diabetics [23].

In a subsequent analysis, the Alpha Omega investigators explored the interactions between statins and n-3 with the hypothesis that statins may have reduced the protective effects of n-3 [39]. They analyzed separately statin users and non-users. Among statin users, 13% developed CVD complications against 15% among non-users and n-3 supplementation did not reduce complications among statin users. However, among statin non-users, only 9% of those receiving n-3 developed CVD complications compared with 18% in the placebo group (hazard ratio 0.46, 95% CI 0.21 to 1.01). The authors conclude that statins reduced the effects of n-3 fatty acids [39]. But contrary to their hypothesis stating that the absence of benefits of n-3 resulted from a dilution effect - no additional benefit of n-3 could be seen in patients strongly protected by statins - it might also be hypothesized that statins inhibit n-3 because the lowest risk was indeed seen among statin non-users taking n-3.

Two other trials [40,41] combining n-3 fatty acids and statins may provide useful information regarding the interactions between n-3 and statins in patients with established ischemic heart disease (secondary prevention).

The first one, named JELIS (for the Japan EPA Lipid Intervention Study), is a very large open-label trial published in 2007 that tested the effect of 1.8 g/day of EPA associated with a statin in patients with or without (74% of the cohort) ischemic heart disease [40]. The control group was receiving a statin only (no placebo). The authors report a significant effect of EPA on the primary endpoint. In a secondary analysis, among the patients with ischemic heart disease (n = 4,848), 197 events occurred in the control group after a mean follow-up of 4.6 years against 158 in the EPA group (hazard ratio 0.81, 95% CI 0.66 to 1.00). This non-significant difference between the two groups was not confirmed when only comparing the hard endpoints (cardiac death and nonfatal myocardial infarction) in the two groups. In fact, there were major design issues in this trial (the trial was neither double-blinded nor placebo-controlled) and it is prudent to conclude that high dose EPA had no significant effect in secondary prevention in JELIS [40]. Whether the lack of significant effect resulted from the high consumption of marine n-3 from fish, as expected in these Japanese patients, or from the use of statins or both is not clear.

Finally, the GISSI-HF trial was published in 2008 and reported the effect of 1 g EPA+DHA in patients with chronic heart failure [41]. Most of these patients had mild heart failure (63% were in stage II of the New York Heart Association) and 50% of them had established ischemic heart disease with various degrees of post-infarction left ventricular dysfunction [41]. Thus, about 50% were in the context of secondary prevention of CHD very similar to the patients enrolled in three of the trials discussed above [21-23]. It was, however, a complex protocol with a first randomization, among 7,046 eligible patients, to receive either n-3 or a placebo, and a second randomization among 4,631 of the same cohort to receive either a statin (rosuvastatin) or a placebo. As 778 patients in the n-3 group were receiving a statin before entering the study, 801 in the placebo group, the effect of n-3 was evaluated in 3,098 statin users compared with 3,121 statin users receiving a placebo instead of n-3.

There were, therefore, four subgroups in that trial with one single group receiving only placebos. The investigators published two articles reporting separately the effects of either n-3 [41] or rosuvastatin [42], and ignoring the interactions between the two treatments. The striking and surprising results of these two combined trials were that neither rosuvastatin nor n-3 were protective [41,42]. In GISSI-HF testing n-3, there was a non-significant trend toward protection (9% reduction of mortality, log-rank test *P *= 0.12) but no effect when comparing ischemic complications (fatal and nonfatal myocardial infarction and stroke) with 204 and 208 endpoints in the n-3 and placebo groups, respectively [41]. Strikingly, there was no protection in GISSI-HF testing rosuvastatin [42]. Thus, whatever the endpoints, recurrent infarction, ventricular arrhythmias or recurrent episode of heart failure, no significant protection was observed with either n-3 or rosuvastatin [41,42]. These data were particularly surprising regarding rosuvastatin as, in agreement with the prevalent theory stating that "the higher the risk, the higher the benefits of cholesterol-lowering", these patients should have been protected, in particular those (50% of the cohort) who were in secondary prevention of CHD with various degrees of post-infarct left ventricular dysfunction.

On the basis of GISSI-HF testing either rosuvastatin or n-3, it could be stated that the interactions between rosuvastatin and n-3 resulted in reciprocal inhibition of the statin by n-3 and of n-3 by the statin. However, a similar lack of protection by the statin was confirmed in another trial [43] in which rosuvastatin was tested against placebo (in the absence of n-3) in survivors of a previous myocardial infarction with various degrees of myocardial dysfunction and various symptoms of chronic heart failure. Rosuvastatin was proven again to not be protective [43], including in patients with mild symptoms (stage II of the New York Heart Association Classification) of heart failure, indicating that the lack of effect of the statin in GISSI-HF was not due to any inhibition by n-3. Moreover, it is no longer possible to argue that the absence of benefits of n-3 was due to the striking protection offered by the statin since rosuvastatin was proven to not be protective [42,43].

The two next questions are then whether there are any known biological mechanisms by which statins could inhibit the effects of n-3, thereby supporting the theory that statins inhibit n-3, and why recent RCTs testing statins were negative.

### Mechanisms through which statins may inhibit n-3

Several mechanisms have been identified.

It has been shown, including in an RCT in CHD patients [44], that statins increase arachidonic acid, the main n-6 fatty acid in cell membranes [44,45]. This may in turn inhibit the protective effects of n-3 because n-6 and n-3 fatty acids are in competition through various pathways involved in the development and complications of CHD [3,5,6,12,13,46-48]. Although this view is still discussed [49], n-3 are clearly more protective when n-6 are low [12,13,50] and n-6 might even be deleterious when given in large amounts and in the absence of n-3 [51,52]. Thus statins may inhibit n-3 by interfering in the n-3/n-6 interplay and favoring n-6.

A second mechanism would be through alteration of mitochondrial function, a key component of myocardial preconditioning [53-55]. Chronic myocardial preconditioning, that is, the ability of the myocardium to withstand an ischemia-reperfusion injury and limit the extent of cell death during and after myocardial ischemia [5,6,53,54], is a major determinant of the outcome of any heart attack. Mitochondria are critical in the induction of myocardial preconditioning [55], and also of neuroprotection [56], and n-3 induce a chronic myocardial preconditioning state [5,6] which is likely explained by "improved" mitochondrial function [57-59]. On the other hand, statins are toxic for the mitochondria in a dose-dependent manner [60-62] and patients treated with statins do have impairment of mitochondrial respiration [63]. In animal experiments, coenzyme Q10, a key component in mitochondrial bioenergy transfer and the synthesis of which is inhibited by statins [61], was reduced in case of impaired heart mitochondrial function [61]. Also, in statin-treated dogs, lower coenzyme Q10 was associated with deficits in a task that measures executive function, an equivalent of cognitive function in humans [64,65]. Endogenous production of coenzyme Q10 is inhibited by blocking the HMG-CoA reductase enzyme with statins [61,65] and decreased plasma coenzyme Q10 was confirmed in statin-treated CHD patients included in a RCT [66]. Thus, whereas the precise point of interaction between statins and n-3 and the dose-effect interactions remain to be fully identified, it is clear that n-3 and statins are counteractive at the mitochondria level.

In line with the mitochondria issue discussed above, a growing body of evidence demonstrates a link between disturbances in mitochondrial functioning, insulin resistance and diabetes [67-70]. In particular, mitochondrial function is required for appropriate glucose-induced insulin secretion [67,68]. In addition, statins provoke myalgias [71], often exacerbated by exercise, resulting in reduced physical activity which in turn increases insulin resistance and the risk of type 2 diabetes [72,73]. It is, therefore, not unexpected that statins increase both insulin resistance [74] and the risk of new-onset type 2 diabetes [75-78]. The real incidence and severity of that complication, which increases the risks of fatal diseases, such as cancers, infectious diseases, stroke and myocardial infarction [79], are still unknown. Data extracted from commercial RCTs and *post-hoc *analyses, including meta-analyses of selected RCTs [76-78], do not help to clarify the issue. More convincing data are expected from long-term cohort studies, and one recent study in post-menopausal women reported that statins increased the risk of new-onset diabetes by about 60% [75], which is considerable and needs confirmation. Whether the supposed benefits of statins exceed the diabetes hazard needs careful and independent analysis (see below).

In contrast, n-3, from either plant or marine sources, decrease insulin resistance and the risk of diabetes [80-84]. They interact with the n-3 fatty acid receptor/sensor *GPR120 *[85], whose dysfunction results in insulin resistance and obesity in both rodents and humans [86]. The use of objective biomarkers of n-3 consumption confirmed the (inverse) associations between n-3 and diabetes [87], although confounders may obscure these associations; in particular, the geographic location of the studied populations [88]. This probably reflects the type of fish consumed by the populations, their actual content in EPA+DHA and the presence of environmental contaminants [89]. Actually, exposure to persistent organic pollutants results in mitochondrial dysfunction and increased insulin resistance in both animal and humans [89-92]. Thus, statins and organic pollutants may inhibit the protective effects of n-3 against insulin resistance and diabetes by similarly altering mitochondrial function.

These interactions between statins and n-3 may explain why statins decrease memory [93] and energy and also increase fatigue with exertion [94] since n-3 are major lipids of the brain and nervous system.

Finally, such a negative action on the central nervous system probably explains the confusing data regarding the effects of n-3 in the prevention of cognitive decline because the main negative studies were conducted in patients taking statins [95], whereas the vast majority of the patients in the positive studies were statin non-users [96].

In summary, statins can inhibit the protective effects of n-3 through several established biological mechanisms.

### Why have recent RCTs testing statins been negative?

For some authors statin therapy is the cornerstone of the primary and secondary prevention of CVD [97-100], sometimes even claiming, concerning cholesterol, that "lower is better and physiologically normal" [101], which is controversial. Other experts say that there are very good reasons to "abandon LDL-cholesterol targets" [102]. In view of the weak efficiency of statins in many groups of patients, in terms of absolute risk reduction, for instance in primary prevention [103] and in women [104-107], and because of their (considerably under-estimated) deleterious side-effects [60-65,71,74-78,93,94,108-110], many physicians and experts conclude that it is time to reassess the benefits and risks of statin therapy. Obviously, overestimating clinical benefits or underestimating toxic side-effects is of major importance to public health [111]. And, indeed, studies can be limited by conflicts of interest and results should be interpreted with caution. As an example, most statin RCTs do not report any difference between the placebo and statin groups regarding skeletal muscle toxicity, whereas post-marketing surveillance indicates that at least 15% of statin users do have muscle weakness or pain [112], a side-effect which is dose-dependent and associated with strong impact on quality of life [113]. This clearly indicates that results of commercial RCTs should be taken with precaution.

The discovery that statins inhibit the protection provided by n-3 may be an additional argument for those who think that the use of statins should be restricted. Should then, for example, statins be limited to specific clinical conditions associated with a high absolute CVD risk, such as secondary prevention of CHD, as proposed by certain cardiologists [114]?

Whether the effects of statins are different in secondary and in primary prevention remains a confusing and critical issue in cardiology. Many physicians still think that statins are protective in secondary but not in primary prevention [114]. However, myocardial infarction or stroke in primary prevention results from the same pathological process as infarction (or a stroke) in secondary prevention. The only difference is not the pathophysiology but the level of likelihood in the tested populations: in patients with a prior infarction, the risk is obviously higher than in healthy people without a previous heart attack. The mechanism (thrombotic obstruction, among other possible mechanisms) is the same but, given the very different probability of observing such complications in the two populations, the sample size and duration of follow-up (required to test any hypothesis in a RCT) should be adapted. In brief, we need thousands of healthy people to demonstrate the antithrombotic effect of any treatment but only hundreds of survivors of infarction. The same reasoning applies for the effects of statins and their hypothetic anti-obstructive effect. Thus, if statins are not protective in primary prevention, there is no scientific or medical reason to believe that they are protective in secondary prevention; and it is exactly what we have seen in recent RCTs in both primary and secondary prevention. The best illustration of that are the four RCTs testing rosuvastatin. It is noteworthy that these four placebo-controlled trials were published after the implementation of the new Clinical Trial Regulations [42,43,115,116]. This is a critical issue because investigators and sponsors were then aware that they were under careful surveillance (contrary to the past) and that they had to comply with a complex and demanding set of legal, ethical and regulatory requirements, contravention of which may lead to criminal proceedings [117,118].

The story should be briefly recalled. Since the Vioxx debacle [119,120] and the implementation of the new Clinical Trial Regulations and the Good Clinical Practice Directive 2005/28/EC [117,118], there have been fundamental changes in the conduct and reports of RCTs. Inspections by health authorities now concern study sites, laboratories, sponsors and contract research organizations. Clearly, the prevalence of bias, spin and misreporting in RCTs has significantly decreased [121-124] although confusion and controversies still exist regarding the quality of many studies, as well as the safety and real benefits of many marketed products [125-130]. And indeed, since the implementation of the new Clinical Trial Regulations [117,118], all the RCTs testing the effects of statins in patients at high risk of CVD and expected to get large benefits of cholesterol-lowering, (patients with post-infarct left ventricular dysfunction [42,43], chronic kidney failure [115,131] or diabetes [131-133]) were either negative or sometimes obviously flawed or misinterpreted [116,133-137]. These striking temporal changes in the efficiency of statins tested in RCTs, before and after the implementation of the new Clinical Trials Regulations and improvement of surveillance by the Health Authorities and politicians [42,43,115,131-137], not only raised puzzling questions about the use of statins in high-risk patients but also question the validity of the many positive commercial RCTs published before the new Regulations came into force, that is, before 2006-2007 [133,136,137].

Let us consider the four placebo-controlled trials testing rosuvastatin. They were conducted in patients with post-infarct left ventricular dysfunction [42,43], chronic kidney failure [11] and in primary prevention [116]. Regarding the first two RCTs, CORONA and GISSI-HF [42,43] already discussed above with the RCTs testing n-3, some experts argue that the failure of rosuvastatin to reduce the risk in these specific patients was not surprising because almost no CVD complications or deaths were expected to fall into the category that statins could prevent, for example, myocardial ischemia or infarction and stroke. Actually, as shown in Table 2 in the CORONA article [43], this view is totally wrong, as 588 and 554 ischemic coronary events were recorded in the two groups in CORONA. In addition, 283 and 272 cardiac deaths occurred in ischemic coronary events against only 191 and 193 deaths due to worsening heart failure, a type of death not expected to be prevented by the statin [43]. In other words and in accordance with the cholesterol-statin theory, the risk of most of these ischemic events should have been reduced by the statin treatment. Thus, unexpectedly the statin failed in CORONA [43] despite striking reduction of cholesterol levels as well as the inflammatory marker CRP!

In fact, the large numbers of ischemic complications in CORONA were not unexpected - neither the sponsors nor the investigators were naive enough to launch a very expensive trial without the hope that rosuvastatin will be effective - because 100% of the recruited patients were survivors of a previous myocardial infarction and thus expected to be at high risk of recurrence, the best situation in theory to prescribe a statin and demonstrate its effectiveness. The fact that they also had various degrees of post-infarction left ventricular dysfunction and some symptoms of chronic heart failure does not change the problem, as perfectly understood by the sponsor when launching the trial. As a matter of fact, when looking at the effects of rosuvastatin in function of the severity of the symptoms of chronic heart failure, there was again no difference between the groups: those with mild heart failure (NYHA class II) symptoms also had no reduction of the primary endpoint: 219 vs. 217 events in the placebo group.

GISSI-HF, another RCT testing rosuvastatin, is a little bit different because only 50% of the patients were survivors of a previous infarction and, thus, in secondary prevention [42]. However, regarding the occurrence (or recurrence) of ischemic events (myocardial infarction and stroke), the same trends were observed in GISSI-HF as in CORONA, with a total lack of effect of the statin regarding the ischemic events expected to be prevented by the statin [42].

CORONA and GISSI-HF, the first trials testing statins in secondary prevention since the implementation of the New Clinical Trial Regulation, proved to be negative despite a striking reduction of cholesterol (and of the inflammatory marker CRP), thus raising one major question: did the past "positive" trials - published before the implementation of the New Clinical Trial Regulation, with statins in secondary prevention conform to the present scientific standards? As discussed below about the landmark 4S trial (as an example) in secondary prevention [138], this is very doubtful.

The third RCT testing rosuvastatin against placebo was the AURORA trial in patients with chronic kidney failure [115]. More than 50% of the patients had some cardiovascular diseases as seen in Table 1 in the AURORA article, in addition to their kidney problem, and were, therefore, also in secondary prevention. However, despite striking reduction of cholesterol and CRP, rosuvastatin failed to show any protection; which is in agreement with the results of another RCT (testing atorvastatin this time) and in similar chronic kidney failure patients mixing primary and secondary prevention [131].

The next obvious question is: if the statins are not effective in these high-risk patients (secondary prevention) why would physicians expect them to be efficient in low-risk patients (primary prevention)? This is a critical public health issue and the last rosuvastatin RCT may help answer the question.

Actually, the fourth placebo-controlled RCT testing rosuvastatin was JUPITER in primary prevention [116]. There have been many critiques regarding JUPITER [136,139-141] because of trial design and conduct issues. Among them, it is noteworthy that there have been over the years several versions of the cardiovascular mortality endpoint, the major endpoint in cardiovascular epidemiology, the versions provided by the sponsor to the FDA [142,143] being different from those published in medical journals [116,144,145]. This indicates a weak and confusing clinical endpoint adjudication process. Still more puzzling, we note that two different versions of the overall mortality curves were published by the JUPITER investigators. In the first version in 2008, the Kaplan-Meier curves were converging [116] - indeed noted by the FDA statisticians as a critical issue to interpret the effects of rosuvastatin on mortality [142] - whereas in the second version published in 2009, the curves were consistently and sustainably diverging [145]. Whether these variations in clinical endpoints and survival curves represent misreporting or a flaw is not the point of this article. However, it makes the JUPITER results at least doubtful and not consistent [136,139-145].

In any case, it is now apparent that experts independent from the sponsor should have a look at the raw (hospital) data of each patient, including those who do not have any complication during the trial, before validating (freezing) the dataset and starting the statistical analysis. Only one version of the clinical results should exist and the statisticians should be totally independent from the sponsor and totally free of any conflict of interest. External audit should be an obligation to re-introduce confidence regarding the validity of the datasets of commercial trials.

In that context, what should we think about JUPITER and the different versions of cardiovascular mortality?

Apparently, (we were unable to find any secondary review of JUPITER endpoint by the FDA officers), the Endocrine-Metabolic Division of FDA never challenged or disputed any data the sponsor presented on JUPITER. FDA officers simply accepted the sponsor's JUPITER data as 100% true and without any mistake; although the sole existence of several different versions of cardiovascular mortality should have motivated an independent audit of the raw data obtained in each site.

To summarize, the overall clinical data regarding rosuvastatin, (three totally negative RCTs in high-risk patients mainly in secondary prevention [42,43,115] and one highly questionable trial in primary prevention [116]), suggest that the implementation of the New Clinical Trial Regulations had a major negative impact on the efficiency of statins to reduce the risk of CVD complications. As the only statin tested in these new regulatory conditions was rosuvastatin, the next question is whether other statins would have been as efficient as they have been reported to be if they were tested in the same regulatory conditions as rosuvastatin. The answer is likely in the recent SATURN trial in which two intensive statin regimens, one of them being rosuvastatin (40 mg daily) and the other one atorvastatin (80 mg daily), were compared [146]; there was no between-group difference in the numbers of CVD complications recorded during this short trial (52 and 49 events) as well as for the surrogate ultrasound endpoint [146]. This total absence of difference between the two statins in SATURN actually suggests that, after the implementation of the New Clinical Trial Regulations, the (lack of) effect of atorvastatin parallels the (lack of) effect of rosuvastatin. This is not unexpected given the parallel total absence of effects of both atorvastatin and rosuvastatin in patients with chronic kidney disease as discussed above [115,131].

This raises major questions regarding the possibility of summarizing and encompassing the totality of the data about statins. Incorporating in the same meta-analysis RCTs published before and after the implementation of the New Clinical Trial Regulations [99,103-106,133,135,147,148] is highly questionable, in particular when nonrandomized subgroup (and questionable) data are included into the analysis [99,103-106,133,135,147,148]. In the same way, it could be said that meta-analyses incorporating flawed RCTs and/or not incorporating unpublished and "unknown" RCTs suffer major publication bias and are of low scientific and medical value. It is well established that industry-sponsored RCTs are more likely than non-industry-sponsored trials to report favorable results for drug treatment because of biased reporting, biased interpretation, or both [149]. Also, investigators of the RCTs testing the statins, and reported between 1994 and 2004, were rarely independent from the sponsors and, in some landmark RCTs, such as 4S [138], MIRACL [150] and CARDS [151] (and also in the recent JUPITER trial [116]), the sponsor employees themselves were actually conducting the study on the field or even in charge of the data analysis [138,150]. This is even more problematic than a presumed lack of independence of the investigators from the sponsors and is today, and should have been, unacceptable. Investigators must be totally and unequivocally independent from the sponsors to be credible. In the 4S and MIRACL studies, for instance, the only statistician of the studies was an employee of the sponsor, which raises major concerns regarding the validity of the results of 4S [138,150] as any sponsor's employee would inevitably favor the product of his employer.

The ultimate and critical question therefore is: should we take seriously the results of RCTs reported before the implementation of the New Clinical Trials Regulations?

In the same way, what is the scientific value of the many meta-analyses [76-78,97,99,103-106,135,147,148] pooling data from commercial RCTs published before and after the New Clinical Trial Regulations? The obvious contradiction between the results of past and recent RCTs testing the statins is a major public health issue as the primary explanation is that the past RCTs were not conducted in agreement with the new Clinical Trials Regulations and that their results were probably not scientifically valid.

The same reasoning should apply to meta-analyses summarizing the cardiovascular effects of n-3 supplements by pooling data of past and recent RCTs [24,152].

## Conclusions

In patients taking a statin, n-3 supplements are not effective against CVD complications, including studies in which statins had no effect. This excludes a dilution effect, that is, a lack of additional benefits of n-3 in patients already protected by statins, and suggests that statins actually inhibit n-3.

Although confirmation is needed, such a negative interaction would have major clinical implications while likely explaining, at least partly, the negative results of the most recent RCTs testing n-3 supplements in high-risk patients [18-24]. An additional, not alternative, explanation is that, in contrast with the past trials involving high-risk n-3 deficient patients [10-13], most patients enrolled in recent RCTs were not severely n-3 deficient [18-23]. The risk related to the omega-3 index was, therefore, not high in these patients [18-23] and, in turn, the expected benefits of n-3 supplementation were not high, which may explain that statins could have almost totally eliminated the small benefits expected from n-3 supplements in these specific patients. It is, therefore, not surprising that in the recent ORIGIN trial testing the effects of about 900 mg of EPA+DHA in patients with dysglycemia no benefit was observed [153]. In ORIGIN, more than 50% of the patients were taking a statin, the median intake of EPA+DHA was higher than 200 mg/day, indicating that the overall intake of n-3 was not low and that the patients were not n-3 deficient, and the absolute risk of CVD complications (< 3 per 100 patients-year) was low [153].

Thus, in agreement with the omega-3 index concept, n-3 supplements would be protective only in patients who are more or less n-3 deficient and not in patients who are at high risk for reasons other than n-3 deficiency.

In other words, n-3 are indispensable nutrients, which means that it is dangerous to be n-3 deficient, which is not rare in many populations [1,2,14], and not drugs susceptible to reducing the overall risk of CVD complications independent of the n-3 status of the persons studied.

As discussed in the Introduction section, this view does not include clinical conditions other than CVD. For instance, the right dosage of n-3, in the absence of statins, for the prevention of cognitive decline and dementia might be much higher than the current recommended dietary intake to prevent CVD [14,38]. Further studies are needed to answer this question. The design (and results) of future RCTs testing n-3 supplement should take that issue into consideration.

In conclusion, the present analysis raises several major questions regarding the optimal strategy to prevent the development and complications of CVD.

Given the weak (or lack of) efficiency of statins in recent RCTs and their major toxic side-effects, including inhibition of n-3, what should physicians do?

The priority is to adopt a healthy lifestyle, which is the critical strategy to be actually protected [12,13,54,72,73,84,92,154,155]. Should physicians continue to prescribe statins?

Because of the many insidious side-effects of statins and the lack of independent recent data confirming the benefits of statins in both primary and secondary prevention [136,137], we actually need a new and independent re-evaluation of the benefit/risk ratio of statins.

In contrast, and given the almost total innocuousness of n-3 in most populations, n-3 supplements should be given without restriction to any patient potentially n-3 deficient and systematically in all patients with established n-3 deficiency. This will give time to change the dietary habits - the alternative solution to correct any degree of n-3 deficiency [12-14,38,54,80-84] - of these patients at high risk of fatal CVD complications because of n-3 deficiency.

Definitely, it is time to rethink the use of n-3 supplements and statins (and other cholesterol-lowering drugs) for the prevention of CVD complications. Only scientists and physicians free of conflicts of interest and independent from the pharmaceutical industry, both the n-3 supplement and statin industries, should be invited to review the whole story from the beginning.

## Abbreviations

ALA: alpha-linolenic acid; CHD: coronary heart disease; CVD: cardiovascular disease; DHA: docosahexanoic acid; EPA: eicosapentanoic acid; ICD: implantable cardiac defibrillator; n-3: omega-3 fatty acids; n-6: omega-6 fatty acids; RCT: randomized controlled trial.

## Competing interests

The authors declare that they have no competing interests.

## Authors' contributions

MdeL drafted the manuscript. PS, PD and MR critically revised the manuscript. All authors have read and approved the manuscript for publication.

## Pre-publication history

The pre-publication history for this paper can be accessed here:

http://www.biomedcentral.com/1741-7015/11/5/prepub
